# Analysis of difficulty in placement of pulmonary artery catheter through the left internal jugular vein

**DOI:** 10.1186/s40981-020-00366-z

**Published:** 2020-08-14

**Authors:** Akiko Tomita, Shoko Takada, Tomoko Fujimoto, Mitsuo Iwasaki, Yukio Hayashi

**Affiliations:** 1 Anesthesiology Service, Sakurabashi-Watanabe Hospital, Osaka, Japan; 2 Present address: Anesthesiology Service, Osaka City General Hospital, 2-13-22 Miyakojima-hondori, Miyakojima-ku, Osaka, 531-0021 Japan; 3 Department of Anesthesiology, Osaka University Hospital, Suita, Osaka, Japan

**Keywords:** Pulmonary artery catheter, Difficult placement, Left internal jugular vein, Tricuspid valve

## Abstract

**Purpose:**

The left internal jugular vein may be an alternative route for the placement of a pulmonary artery catheter when the right jugular vein is not available. Although the placement through the left internal jugular vein is expected to be more difficult, little has been written regarding difficulties in achieving proper placement of the catheter through the left internal jugular vein.

**Methods:**

This prospective and observational study includes patients undergoing cardiac surgery with the catheter placement by monitoring the pressure waveform for 2 years. We measured the time required for the catheter to pass through the tricuspid and pulmonary valves, respectively. The data were analyzed by Mann-Whitney. *P* < 0.05 was considered significant.

**Results:**

The catheter placement through the right and left internal jugular vein was done in 285 (group R) and 10 patients (group L), respectively. The time duration through the tricuspid valve in group L was significantly longer than that in group P (8 [5–14] s vs 70 [19.8–138] s, median [range], *P* < 0.01), whereas the time duration through the pulmonary valve was comparable between the two groups (15 [10–27.75] s vs 15 [10.25–19] s, median [range], *P* = 0.62).

**Conclusion:**

These results indicate that the difficulty in the catheter placement through the left jugular vein may be to pass through the tricuspid valve, not the pulmonary valve.

## Introduction

Pulmonary artery catheter (PAC) is an established monitoring device for anesthetic management of patients undergoing cardiac surgery [1], although a recent study showed that PAC should not be used routinely for patients with a low risk of hemodynamic disturbance [2]. It is universally accepted that PAC should be placed through the right internal jugular vein from an anatomical point of view [3, 4]. On the other hand, the left internal jugular vein would be an alternative route if this vein were not available for certain reasons [4]. One may claim that it is no wonder that the placement through the left internal jugular vein may be more difficult than that through the right jugular vein. However, to our knowledge, there has been no clinical data regarding difficulties in achieving proper placement of PAC through the left internal jugular vein in comparison with the right internal jugular vein. Thus, we would like to formally evaluate what is considered to be the common notion.

In the last 2 years (2017, October ~2019, October), we placed a PAC in 301 patients undergoing cardiovascular surgery after anesthesia. Among those patients, we encountered 10 patients whose PAC placement was intentionally done through the left jugular vein. This prospective observational study was designed to compare difficulties during the PAC placement through the left jugular vein to placement through the right jugular vein.

## Method

This prospective observational study was approved by the institutional review board and registered in the UMIN Clinical Trial Registry (UMIN 27418). Written informed consent was obtained from all eligible patients. This study was conducted from October 2017 to October 2019 at Sakurabashi-Watanabe hospital in Osaka, Japan. We prospectively examined the time required for the PAC placement in 301 adult patients undergoing elective cardiovascular surgery. Patients who had a history of tricuspid ring annuloplasty or tricuspid valve replacement were excluded [5]. After anesthesia, the PAC (continuous cardiac output/SvO_2_ Catheter 744HF75, Edwards Lifesciences, Irvine, CA, USA) was inserted through the right or left internal jugular vein. First, the introducer sheath was placed via the internal jugular vein in the Trendelenburg position, and then, the PAC was started floating through the sheath by monitoring the pressure waveform in the flat position. The PAC was inserted until the central venous pressure (CVP) waveform was confirmed, and subsequently, the balloon was inflated with 1.5 ml of air. With an inflated balloon, the catheter floated into the pulmonary artery (PA).

The time required for PAC to pass through the tricuspid and pulmonary valve was measured. The catheter passage time for the tricuspid valve and the pulmonary valve was defined as the duration of time required for the catheter to float from the CVP position through the tricuspid valve to the right ventricle, and that from the right ventricle through the pulmonary valve to the pulmonary artery, respectively. That is, the first beginning time point was just after the inflation of the balloon to start floating the catheter, and the first ending time point was the time when we first observed the waveform of the right ventricle. The second beginning time was to restart floating the catheter with the waveform of the right ventricle, and the second ending time point was the time when we first observed the waveform of the PA. If the placement failed to precede the catheter into the PA within 5 min, we regarded this case as a failure and some guidance such as transesophageal echocardiography or X-ray fluoroscopic system was used.

We classified the patients into two groups depending on the internal jugular vein we used, that is, the right internal jugular vein (group R) or the left internal jugular vein (group L). The patient’s characteristics and clinical data were collected from their medical record.

We had no a prior knowledge of the variability of time required for the catheter to pass through the tricuspid and pulmonary valves in our anticipated population of cardiovascular surgery candidates. Thus, considering that our record showed there were three cases whose PAC placement was intentionally done through the left jugular vein for 1 year before the present study period (2016, October ~2017, October), we planned to collect data for 2 years and then to perform the sample size calculation. Accordingly, our sample size scheduled was arbitrary and was determined entirely by the number of eligible and accessible patients during the time available to execute this study. Actually, we collected data of the PAC placement through the right internal jugular vein in 285 patients and those through the left internal jugular vein in 10 patients for the 2-year study period. As a result, we found a significant difference in the time duration to advance the PAC through the tricuspid valve. So, no formal sample size calculation was performed and we decided to present these 2 years data for analysis.

Data were expressed as means ± SD or a median and interquartile range as appropriate. Patient’s characteristics were analyzed by unpaired *t* test, the time duration through the tricuspid valve and the pulmonary valve was compared by Mann-Whitney, and the success rate of the PAC placement was expressed in percentages and analyzed by *χ*^2^ test. *P* < 0.05 was considered statically significant.

## Results

There were five patients with a history of tricuspid ring annuloplasty or tricuspid valve replacement, and one patient whose data were missing. Therefore, 295 patients were included in this analysis. We performed the PAC placement through the right internal jugular vein in 285 patients (group R) and through the left internal jugular vein in 10 patients (group L). The reason why the left internal jugular vein was selected in these patients was that there was a CVP catheter or a pacing lead already placed through the right internal jugular vein, or stenosis in the right internal jugular vein. The patient characteristics of both groups are shown in Table 1. No significant differences existed in age, sex, height, weight, body mass index, or preoperative cardiac function including the degree of tricuspid regurgitation between the two groups, although emergent operation in group L was more frequent than in group R (*P* = 0.039). The catheter could not be placed within 5 min in three patients in group R, whereas the placement of a PAC was successful in all of 10 patients in group L (Table 2). The time duration to advance the PAC through the tricuspid valve in group L was significantly longer than that in group R, while the time duration for the pulmonary valve was comparable between the two groups (Table 2).

**Table 1:** Patient characteristics and clinical data

	Group R (*N* = 285)	Group L (*N* = 10)	*P* value
Age (year)	68.5 ± 11.6	74.8 ± 6.3	0.083
Sex/men (%)	187 (65.6)	8 (80)	0.503
Height (cm)	161.7 ± 10.7	159.6 ± 5.8	0.302
Weight (kg)	61.3 ± 12.4	58.7 ± 23.3	0.084
Body mass index (kg/m^2^)	23.3 ± 3.4	22.7 ± 7.2	0.140
Ejection fraction (%)	62.2 ± 13.8	51.2 ± 19.7	0.135
Cardiothoracic ratio (%)	52.5 ± 6.9	53.0 ± 5.8	0.710
Tricuspid regurgitation (degree)	1 (0 - 1.3)	2 (1 - 2)	0.131
Atrial fibrillation (%)	33 (11.6)	2 (20)	0.337
PM/CRTD implantation (%)	11 (3.9)	2 (20)	0.066
Redo cardiac surgery (%)	28 (9.8)	1 (10)	0.999
Emergent surgery (%)	21 (7.4)	3 (30)	0.039

Values are expressed as mean ± SD, median (interquartile range), or the number of patients (%)

*Group R* right internal, *group L* left internal jugular vein, *PM* pacemaker, *CRTD* cardiac resynchronization therapy defibrillator

**Table 2:** Successful rate and the time duration to advance the pulmonary artery catheter through the tricuspid and pulmonary valves

	Group R (*N* = 285)	Group L (*N* = 10)	*P* value
*N* (success/failure)	282/3	10/0	
Successful rate (%)	98.9	100	0.999
Time duration (s)			
Tricuspid valve	8 (5–14)	70 (19.8–138)	< 0.001
Pulmonary valve	15 (10–27.75)	15 (10.25–19)	0.620

Values (time duration) are presented as median (quartile)

*Group R* right internal, *group L* left internal jugular vein

## Discussion

The principal finding of this study is that placement of a PAC through the left internal jugular vein by monitoring the pressure waveform needed a longer time to cross over the tricuspid valve than the placement through the right internal jugular vein, while the time to cross over the pulmonary valve was comparable.

Anatomically, it has been well known that it may be difficult to approach from the left internal jugular vein to the superior vena cava, because the venous course forms the left internal jugular vein arcs through the innominate vein to the superior vena cava in a gentle curve, while the right internal jugular vein follows to the superior vena cava directly [3]. This longer and more complicated route to reach the tricuspid valve from the left internal jugular vein may be related to the present results, that is, the longer time was needed to pass the tricuspid valve. The present study was designed to place a PAC by monitoring the pressure waveform, and we started the measurement after the CVP waveform was confirmed. Thus, we have to acknowledge that the CVP waveform may not always guarantee that the tip of the catheter exists in the right atrium especially in group L. So, it may be possible that the catheter migrated to a wrong vein, such as right internal jugular vein and internal thoracic vein, or made a loop in the right atrium. With helpful visual guidance including transesophageal echocardiography and X-ray fluoroscopic system, we could identify the location of the catheter and reduce the time duration in group L. In fact, recent reports recommended using transesophageal echocardiography in potentially difficult patients such as those with pulmonary hypertension [6, 7].

Thus, one may deduce that the present results showing that a longer time was needed to advance a PAC through the tricuspid valve in group L is as expected. On the other hand, it is interesting that the time duration to pass through the pulmonary valve was comparable, for it suggests that difficulty of the catheter placement to the pulmonary artery is similar regardless of the cannulation sites, once a PAC can pass through the tricuspid valve and reach the right ventricle.

It is generally accepted that visual guidance is preferred to monitoring the pressure waveform. Nevertheless, the placement of a PAC with the pressure monitoring is still common in clinical situations. Presumably, these guidance systems need additional factors, such as manpower and exposure of radiation. In addition, a recent report has documented that placement with monitoring the pressure waveform is not time-consuming compared with transesophageal echocardiographic guidance [7]. Thus, if we try placement of a PAC through the left internal jugular vein with monitoring the pressure waveform and feel difficult to cross over the tricuspid valve, we should not hesitate to induce visual guidance to pass the tricuspid valve. On the other hand, once a PAC can pass the tricuspid valve, we may be able to advance the catheter to the pulmonary artery without difficulty.

This was designed to be a prospective and observational study in the course of 2 years, so the number of group L was exclusively small and characteristics of the patients in each group were not completely identical (Table 1). If a strict comparison of the two cannulation sites was planned, a randomized control study would be preferred. However, it is clinically established that the right internal jugular vein is the preferred site for cannulation and the left jugular vein is less reliable [4]. Therefore, a randomized control study to compare the right and left internal jugular vein might be unacceptable from an ethical point of view. We suppose that our study design may be an alternative option, and we acknowledge that the clinical significance of the present results would be interpreted with caution.

We have to discuss potential limitations in our study. This study included multiple anesthesiologists who performed placement of a PAC, so the technique of the placement may not be identical [8]. Thus, these individual differences might affect the results of this study.

## Conclusion

In conclusion, when the left internal jugular vein was chosen for the cannulation site, the catheter passage time for the tricuspid valve was significantly longer than that using the right internal jugular cannulation, while the catheter passage time for the pulmonary valve was comparable with either cannulation site.

## Data Availability

The data that support the findings of this study are available from the corresponding author on reasonable reason.
